# Assessment of the Cyclic Fatigue Performance of the Novel Protaper Ultimate File System Used in Different Kinematics: An In Vitro Study

**DOI:** 10.3390/jfb15040085

**Published:** 2024-03-29

**Authors:** Cezar Tiberiu Diaconu, Anca Elena Diaconu, Mihaela Jana Tuculina, Laurența Lelia Mihai, Mircea Gheorghiță, Lelia Mihaela Gheorghiță, Petre Mărășescu, Alexandru Gliga, Oana Andreea Diaconu

**Affiliations:** 1 Faculty of Dental Medicine, Department of Endodontics, University of Medicine and Pharmacy of Craiova, 200349 Craiova, Romania; diaconu_czr@yahoo.com (C.T.D.); anka.diaconu@gmail.com (A.E.D.); leliagheorghita@yahoo.com (L.M.G.); oanamihailescu76@yahoo.com (O.A.D.); 2 Faculty of Dental Medicine, University Titu Maiorescu of Bucharest, 031593 Bucharest, Romania; 3 Faculty of Dental Medicine, Department of Oral and Maxillofacial Surgery, University of Medicine and Pharmacy of Craiova, 200349 Craiova, Romania; gheorghitamircea@yahoo.com; 4 Faculty of Dental Medicine, Department of Dental Prothesis Technology, University of Medicine and Pharmacy of Craiova, 200349 Craiova, Romania; marasescup@yahoo.com; 5 Faculty of Dental Medicine, Department of Operative Dentistry, “Carol Davila” University of Medicine and Pharmacy, 050474 Bucharest, Romania; dr.alexandrugliga@gmail.com

**Keywords:** endodontics, cyclic fatigue, NiTi alloy, forward reciprocating motion

## Abstract

This in vitro study aims to assess the cyclic fatigue resistance of the Protaper Ultimate (PTU) files compared to the Protaper Gold (PTG) and the M3 UDG (M3) files using various motion kinematics in simulated canals. As far as the authors are aware, no study has previously compared the three file systems before this current investigation. Therefore, closing this information gap is the goal of the current research. Methods: A total of (60 new endodontic files were randomly divided into 6 groups (10 files per group); groups 1, 3, and 5 used continuous rotation (CR), while groups 2, 4, and 6 used forward reciprocating motion (FRM). A manufactured stainless-steel artificial canal was used to perform the cyclic fatigue testing. The results were analyzed using Student’s *t*-test and two-way ANOVA. All pairwise comparisons revealed statistically significant differences in the time to failure (TTF) for every study group (*p* < 0.001), with the exception of the PTG and M3 files, which performed similarly using both CR and FRM. Conclusions: The PTU files performed better than the PTG and M3 files in terms of the TTF and number of cycles to failure (NCF) using both CR and FRM.

## 1. Introduction

The primary goal of successful endodontic therapy is to perform a proper cleaning and shaping of the root canal system. In clinical practice, this cleaning and shaping, performed with the aid of endodontic files manufactured from stainless steel or nickel–titanium (NiTi) alloy, is not always easy due to the complex anatomy of the root canal system. One of the most problematic situations that a clinician can encounter during root canal therapy is file breakage inside the root canal, hence the need to develop more elastic and resilient file systems. Manufacturers have been constantly developing and improving the alloy and design of the file systems since the first introduction to clinical practice of stainless-steel files that were consequently outperformed by NiTi files [1]. Compared to the traditional stainless-steel endodontic instruments, the more modern NiTi alloy endodontic instruments present enhanced flexibility and strength characteristics [2], which make root canal therapy easier by enhancing the speed, precision, and safety of root canal shaping [3]. During root canal shaping, an unanticipated instrument fracture may arise due to torsional failure or cyclic fatigue failure, as a portion of the file is subjected to cycles of compression and stretching in the region of the maximal root canal curvature [4,5]. These tensions cause the instrument structure to disintegrate, which ultimately results in fracture [6,7].

Of these two causes, the main reason for NiTi instrument separation is thought to be cyclic fatigue, particularly in canals with abrupt curvatures, where the instrument rotates under greater stress at the point of maximum curvature [8,9]. One way of avoiding this is to employ reciprocating motion. According to the literature, compared to continuous rotation (CR), a reciprocating motion increases the endodontic instruments’ resilience to cyclic fatigue during instrumentation by operating continuously under the elastic limit of the instruments [10,11,12]. All rotary systems, with the exception of reciprocating instruments, are made to cut in a clockwise (CW) direction. If one attempts to employ normal CR cutting instruments using a reciprocating motion, there is a chance that the rotary instrument would not be able to penetrate or cut the canal walls.

Classical reciprocating file systems feature a larger counter-clockwise (CCW) angle of motion than the CW angle because they are intended to cut in a CCW direction [13]. It may be possible to employ traditional rotary glide path and file systems in a reciprocating motion when the CW rotation is greater than the CCW motion since the instrument is cutting in a CW direction, as the flutes of most conventional systems are designed to be operated in continuous CW rotation [14]. 

Although the classical reciprocating motion has been intensely studied, there is only limited information about the effect of forward reciprocating motion (FRM) on the lifespan of the instruments. Our study design aims to fill this information gap and provide a more in-depth analysis of the FRM and the cyclic fatigue resistance of endodontic rotary instruments when this motion is employed.

Researchers have employed a number of cyclic fatigue test models, all of which can provide a curved rotational trajectory. Through cyclic fatigue testing, variables such as the instrument’s size and metal alloy characteristics, the test temperature, kinematics, the handpiece’s static versus dynamic motions, the canal radius, and the degree of curvature have all been identified as potentially affecting the fatigue strength outcomes [15]. Novel finite element models are currently used to study the mechanical performance of endodontic files due to the advancements in computer-aided software; with this approach, although we can use mathematical analyses to compare and address the mechanical performance [16] and stress distribution on the files during root canal therapy [17], we cannot fully understand the metallurgical properties and alloy treatments, which are crucial to the resistance of the file itself. In cases where we perform a comparative study regarding instruments that are very similar in terms of the cross section, geometrical design, pitch angle, and taper, although finite element analysis is a very effective and affordable method [18], it does not take into consideration the metallurgy of the files; hence, we believe that it would not be the best option for testing, and more accurate results will be obtained using a more conventional way of testing cyclic fatigue resistance. As the Protaper Ultimate (PTU) file system claims to be a further development of the Protaper file series, we consider it necessary to fill in the information gap and test the files’ behavior both using CR and FRM in comparison with other file systems, while also taking into consideration that, in the majority of the existing literature in the endodontic field, metallurgy research was not considered when comparing endodontic instruments; instead, mechanical evaluation was the only method used, which led to a constrained result, an incomplete understanding of the subject, and occasionally to inaccurate conclusions. We believe that for a more accurate comparison between various instruments, at least one metallurgical test is necessary in order to enhance the quality of the research and ensure a better understanding of the topic.

### Aim

In this context, the purposes of this present in vitro study were to compare the cyclic fatigue resistance of PTU F2 files with that of the M3 and the PTG F2 files and to evaluate the effect of using FRM on the lifespan of the tested instruments in simulated canals compared to CR.

The null hypothesis was that the cyclic fatigue performance of these files would not differ and that the different motions in which the files were operated would have no effect on the lifespan of the tested instruments.

## 2. Materials and Methods

### 2.1. Study Design

Sixty (60) 25 mm long, new, and unused endodontic rotary files with a 250 µm apical diameter were used in this in vitro study. All NiTi instruments were first inspected under magnification (Zeiss ProErgo, Zeiss, Oberkochen, Germany), and none were discarded.

The following study groups were established.

Group 1 25.08 F2 ProTaper Ultimate (Dentsply Maillefer, Baillagues, Switzerland), (PTU), (*n* = 10), used in continuous rotation;
Group 2 25.08 F2 ProTaper Ultimate (Dentsply Maillefer, Baillagues, Switzerland), (PTU), (*n* = 10), used in forward reciprocating motion;
Group 3 25.08 F2 ProTaper Gold (Dentsply Maillefer, Baillagues, Switzerland), (PTG), (*n* = 10), used in continuous rotation;
Group 4 25.08 F2 ProTaper Gold (Dentsply Maillefer, Baillagues, Switzerland), (PTG), (*n* = 10), used in forward reciprocating motion;
Group 5 25.08 M3 UDG (United Dental Changzhou, Changzhou, China), (M3), (*n* = 10), used in continuous rotation;
Group 6 25.08 M3 UDG (United Dental Changzhou, Changzhou, China), (M3), (*n* = 10), used in forward reciprocating motion.

### 2.2. Testing Device

**Figure 1:**
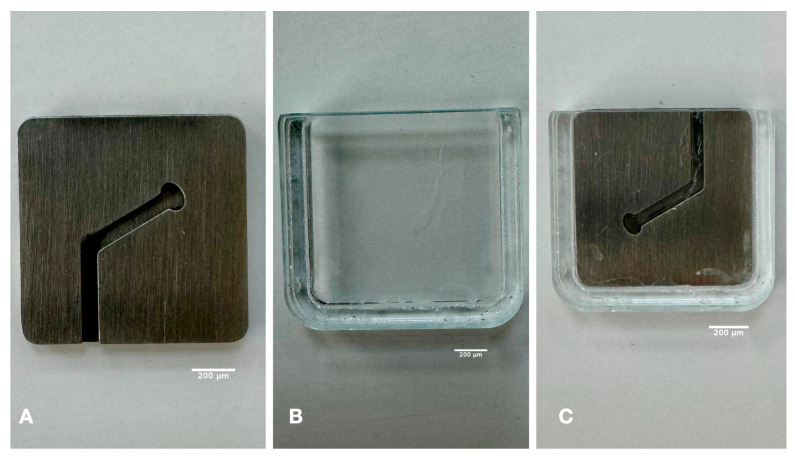
Testing unit: (**A**) artificial canal made of stainless steel using a CNC machine, (**B**) polycarbonate cover, (**C**) artificial canal with polycarbonate cover.

A specially constructed device, which is an advancement over the cyclic fatigue device previously reported in other studies [19] and founded on the cyclic fatigue device created by Plotino et al. [20], was used to conduct the static cyclic fatigue test. An instrument contained in a curved canal can be repeatedly simulated with this curved artificial canal made out of stainless steel with a CNC (Computer Numerical Control) machine. It had an inner diameter of 1.5 mm, a 70° angle of curvature, a 2 mm curvature radius, and a 19 mm length (Figure 1A). The maximum curvature was situated 10 mm from the coronal part of the artificial canal.

The testing unit was also provided with a tight seal see-thru cover (Figure 1B) produced out of transparent polycarbonate to prevent any unwanted slippage of the files from the artificial canal and to maintain and form a reservoir with the artificial canal (Figure 1C) for the high-flow synthetic oil necessary to reduce the file’s friction as it came into contact with the artificial canal walls.

**Figure 2:**
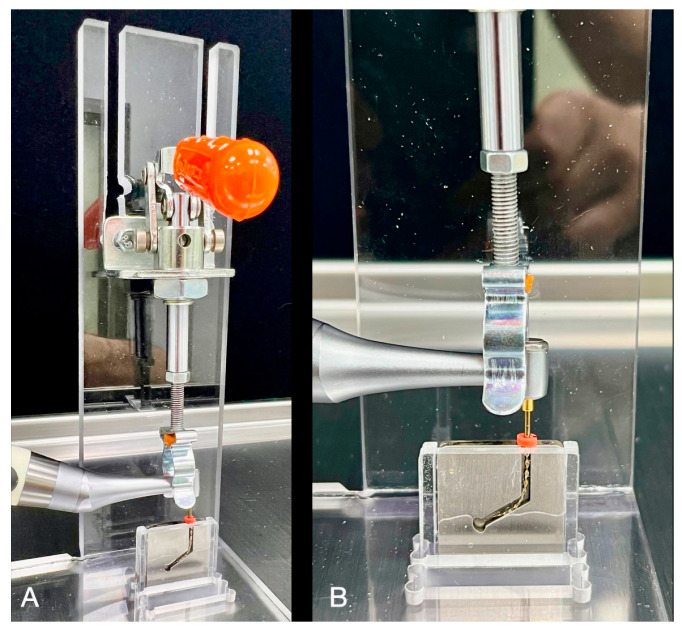
Testing device. (**A**) Global view of the testing device and (**B**) specially designed jig, which held the endodontic motor in a repeatable position with an instrument inserted in the testing unit.

The cyclic fatigue testing unit with its cover was mounted in a fixed vertical position on a testing platform composed of a horizontal polycarbonate plate for the aforementioned testing unit and a vertical polycarbonate plate housing the endodontic motor with the aid of a specially designed jig, which held the endodontic motor in a repeatable position that only allowed the endodontic motor to move in a predetermined vertical manner. The jig was fastened to the vertical polycarbonate plate of the testing platform using two screws (Figure 2A,B).

### 2.3. Testing Conditions

After the polycarbonate cover was placed over the testing unit, to decrease the file friction as it came into contact with the artificial canal walls, the artificial canal was filled with high-flow synthetic oil (Singer All-Purpose Oil; Singer Corp., Barcelona, Spain) for lubrication before each test [21,22]. Each of the files tested was mounted in the endodontic motor and placed into the artificial canal perpendicular to the orifice at the same working length (18 mm).

At room temperature (23 ± 1 °C), the instruments were rotated in the artificial canal using a torque-controlled motor (E Connect S, Eighteeth, Changzhou, China) keeping in mind the recommended speeds and torques of the manufacturer, i.e., 400 rpm and 4 Ncm for the CR (groups 1, 3, and 5). Groups 2, 4, and 6, designated for the FRM, were operated as such.

The instrument rotates both CW and CCW, but the CW motion angle is greater than the CCW motion angle—for example, 90° CW and 30° CCW. It is advised that the CW rotation should be between 90° and 270°, and the CCW rotation should be between 30° and 90°, such that the net CW rotation in each cycle varies between 60° and 240°, resulting in a full 360° CW revolution after 1.5 to 6 cycles. As such, the CW rotation was set to 90°, and the CCW was set to 30°. This setting allowed the files to make a full 360° CW rotation in 6 cycles. For the FRM, the rotational speed and torque were kept the same as for the CR, i.e., 400 rpm and 4 Ncm.

Each instrument was placed into the artificial canal at a working length of 18 mm, and it was rotated there until its fracture. In order to determine the precise TTF, filming and direct observation of the fracture instance were used.

With the use of a digital chronometer, the TTF for each instrument was measured in seconds (s) from the moment it began to operate inside the testing unit until a fracture occurred. The timing stopped as soon as a fracture was audibly or visually identified. 

The average TTF value per group was determined by adding the TTF from each instrument, thus obtaining a sum that was then divided by 10 (number of instruments per group). The following formula was used to convert the average TTF to the number of cycles to fracture (NCF): TTF (s) × rotational speed (rpm)/60. 

### 2.4. Stereomicroscope and X-ray Fluorescence Analysis

**Figure 3:**
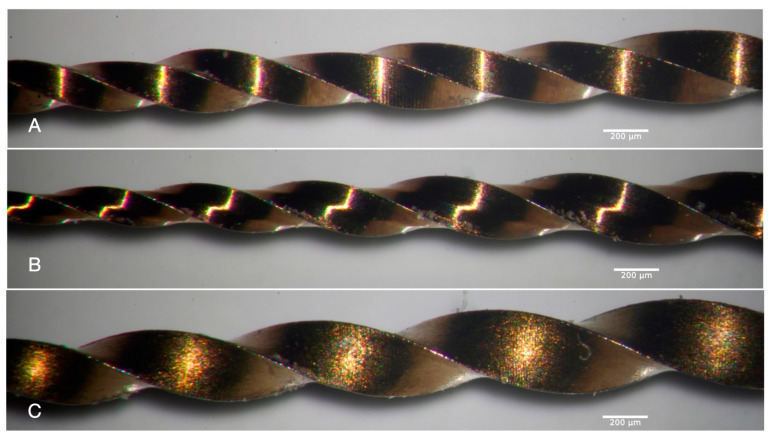
Dental surgical microscope photographs ((**A**) PTU, (**B**) PTG, (**C**) M3) of the instruments before cyclic fatigue testing.

Before being subject to the cyclic fatigue tests, all instruments were initially analyzed using a dental surgical microscope at a magnification of × 15 (Zeiss Opmi Proergo, Zeiss, Oberkochen, Germany) in order to carry out a surface analysis to be able to discard any instrument with preexisting surface defects in its manufacture (Figure 3). All sets of instruments had symmetrical spirals, no defects, and similar surface finishing.

**Figure 4:**
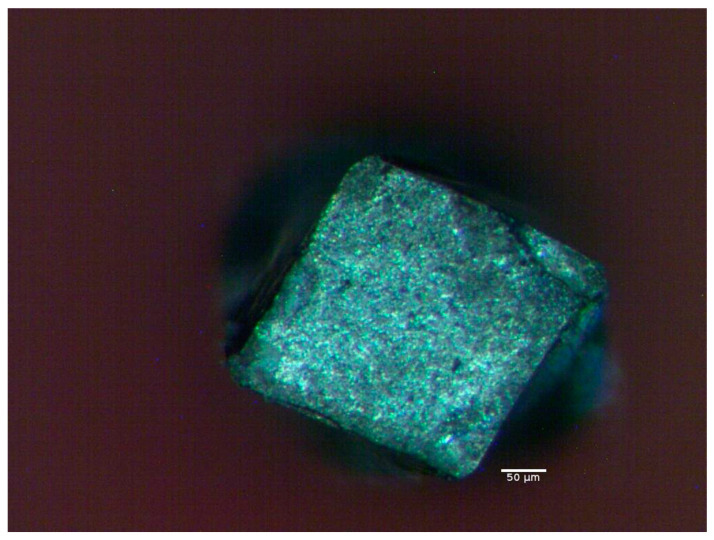
Example of photomicrographs of the fractured surface from the PTU F2 file.

After the cyclic fatigue tests, the separated fragments were cleaned of debris using 70% ethyl alcohol in an ultrasonic bath for 10 min. Three separated fragments from each group were randomly selected and examined using a stereomicroscope (Olympus SZX 7, Olympus Corporation, Tokyo, Japan), and photomicrographs of the fractured surfaces were taken in order to confirm that the cause of the fracture was cyclic fatigue (Figure 4).

**Figure 5:**
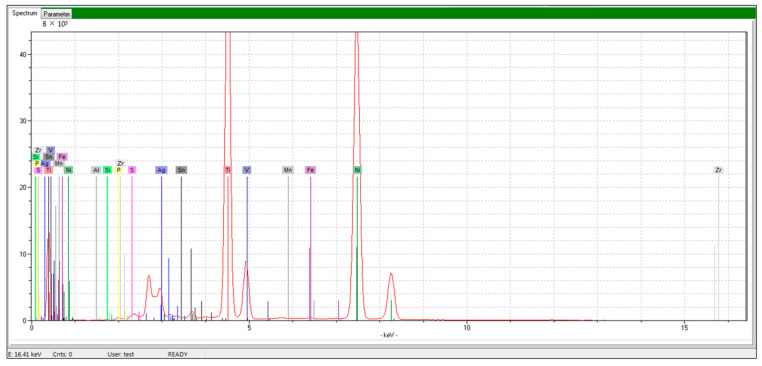
Example of X-ray fluorescence analysis from the PTU files.

In addition, before being subjected to cyclic fatigue testing, all the instruments were studied with the help of an X-ray fluorescence analyzer (XRF) (Bruker S1 Titan, Bruker Daltonics, Bremen, Germany) using a 4 W tube at a tension of 6–50 kV with an intensity of 5–200 mA and the time set at 60 s in order to analyze the metallurgical composition of the key elements of the NiTi instruments, as seen in Figure 5.

### 2.5. Statistical Analysis

Microsoft Excel (Microsoft 365 version 2402, Microsoft Corp., Redmond, WA, USA) and the XLSTAT add-on version 2022.4.5 for MS Excel (Addinsoft SARL, Paris, France) were used for statistical analysis. Excel was used to conduct a descriptive analysis of the study group based on a number of factors (such as the calculation of the mean and standard deviation, two basic statistical indicators). Complex statistical tests (Student’s *t* and ANOVA tests) were performed using the XLSTAT add-on version 2022.4.5. Numerical data were explored for normality by checking the data distribution, and because almost all numerical variables had a Gaussian distribution, we were able to use the parametric tests, Student’s *t*-test and two-way ANOVA. The data were represented by the mean and standard deviation (SD) values. The significance level was set at *p* ≤ 0.05 within all tests.

## 3. Results

When analyzing the performance of each type of instrument using CR versus FRM, using Student’s *t*-test, we found that, for all types of instruments, there were highly statistically significant differences (p Student < 0.001) in regard to the TTF/NCF: p Student = 1.49 × 10^−8^(PTU), p Student = 5.86 × 10^−14^(PTG), p Student = 5.79 × 10^−11^(M3), as shown in Table 1. All instruments showed a statistically significantly improved performance in the cyclic fatigue resistance when FRM was employed in comparison with CR.

When comparing the performance of the obtained values for the TTF/NCF of the three types of instruments using CR, based on a two-way ANOVA test, we found statistically significant differences between the PTU and M3 (*p* < 0.0001) and the PTU and PTG (*p* < 0.0001); however, no statistically significant differences were observed between the TTF/NCF of the PTG and M3 (*p* = 0.998). When comparing the performance of the obtained values for the TTF/NCF of the three types of instruments using FRM, based on a two-way ANOVA test, we found statistically significant differences between the PTU and M3 (*p* < 0.0001) and the PTU and PTG (*p* < 0.0001); however, no statistically significant differences were observed between the TTF/NCF of the PTG and M3 (*p* = 0.323), as shown in Table 2.

### 3.1. Stereomicroscope Evaluation

**Figure 6:**
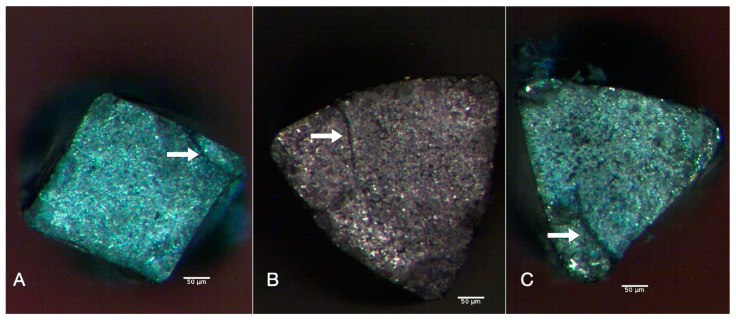
Stereomicroscope photographs of the fractured surface ((**A**) PTU, (**B**) PTG, (**C**) M3). Cracked surfaces marked by arrows.

The stereomicroscope photographs of the broken surface exhibited typical characteristics of cyclic fatigue failure for all instruments. Every instrument displayed microvoids and cracked surfaces, as shown in Figure 6, marked with arrows, which were mechanical signs of ductile fracture.

### 3.2. X-ray Fluorescence Analysis

**Scheme 1:**
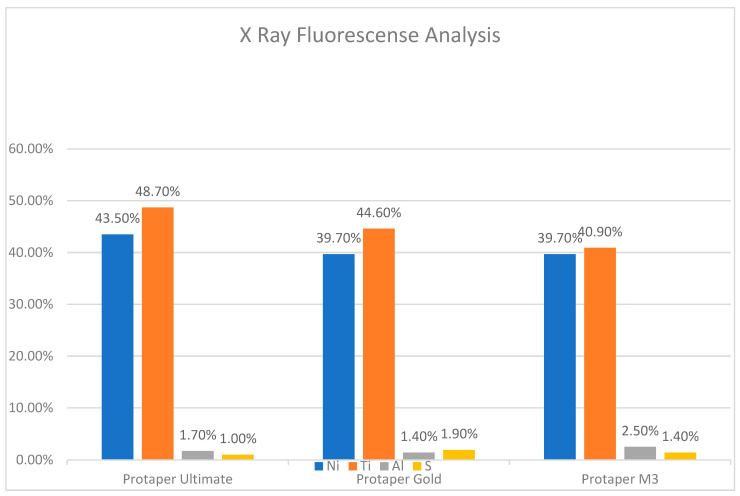
Descriptive X-ray fluorescence analysis of the key elements in the tested instruments.

According to the XRF analysis, which was carried out to verify that the endodontic instruments employed in this present study conformed to the proper standards and specifications and that the chemical composition of the metallic parts had the correct percentage of key elements, we confirmed that the alloy type was NiTi, as seen in Scheme 1.

## 4. Discussion

The cyclic fatigue resistance of the different NiTi files tested was significantly influenced by the kinematics of motion of the NiTi rotary instruments, which led us to reject the null hypothesis that the files’ cyclic fatigue performance would not differ and that the different motions that the files used would have no effect on the lifespan of the tested instruments. 

NiTi instruments were first introduced in 1980 by Walia et al. [23]. Since then, efforts have been made to improve the alloy properties of these files in order to reduce the number of accidents (broken files) that occur during endodontic treatment [7], and a number of factors have been discovered to influence the resistance to cyclic fatigue of any one file, including the pecking motion, which may play an essential role in preventing NiTi rotary file fractures by reducing the file stress in curved canals, which, in turn, lowers the risk of fracture, according to Li et al. (2002) [24]. Before the instruments are subjected to the maximum stressed area once more, the pecking amplitude provides a time buffer in which the files are relieved of stress; therefore, it is advised to use a pecking motion at a low frequency in order to lower the probability of instrument fractures being generated by cyclic fatigue [21]. The pecking motion amplitude and frequency are operator dependent, meaning that in a clinical setting, they may vary from one operator to another; as such, this parameter is more appropriate for in vitro testing. Even if the pecking motion can be standardized in an in vitro setting, this does not mean that the instrument must follow an exact pathway. Taking that into consideration, and in line with other studies that claimed that the static model’s main benefit is the reduction in variables such as the axial movement amplitude [25,26,27], we believe that the best testing approach for this investigation is static testing, which can maintain the instrument at a precise trajectory. Another important factor that can affect the cyclic fatigue resistance of endodontic rotary instruments is the speed at which the endodontic instruments are rotated, as this could play an important role in the cyclic behavior [28]. Various studies have shown that the TTF is linked to the rotation speed, i.e., instruments show more resistance when used at lower speeds [24]; therefore, it is preferable to use instruments at a low speed [29]. In his study from 2014, Pedulla E. [30] found, in agreement with other results, that the rotational speed has no significant influence on the number of rotations needed to fracture NiTi rotary instruments; although speed does not influence the NCF, it has an influence on the TTF, as observed by Zanza A. [31] and Ates et al. (2020) [32], who described an increased NCF but a decreased TTF with XP-endo Shaper instruments (FKG Dentaire, La Chaux-de-Fonds, Switzerland), probably related to the higher speed at which the files were rotated; hence, they were able to perform a larger number of cycles in a shorter period of time; thus, we decided to operate the files involved in this present study at the same speed in order to remove any inconsistencies in the results obtained, i.e., 400 rpm and 4 Ncm.

Research indicates that, independent of the rotational speed, an endodontic instrument’s NCF during in vitro cyclic fatigue tests is influenced by its mass and shape [33,34]. According to Burklein’s 2021 study, the cross-sectional design (i.e., core mass) and the alloy were important variables in instrument failure; however, none of these variables could be identified as the primary parameter for altering the duration and frequency of the fracture cycles. In contrast, it appeared that variations in the time and cycles to fracture were caused by the interplay of several elements (such as the alloy, the longitudinal and cross-sectional design, and the rotational speed) [35]. As a result, in accordance with Burlekin’s study, we found that although the most comparable files from each endodontic rotary system were employed in our study, according to their length, apical diameter, angular movement, speed, and taper, we obtained statistically significant differences (*p* < 0.0001) when comparing the PTU files with the PTG and M3 files using both CR and FRM; however, no statistically significant differences were found when comparing the PTG with the M3 files using CR (*p* = 0.998) and FRM (*p* = 0.323), which leads us to think that the improved performance of the PTU could be linked to the cross-sectional design that is different from its counterparts.

Regarding the increased resistance to cyclic fatigue of all the files tested in our study when FRM was employed, we found, in accordance with previous studies [36], that a decreased reciprocation range of instruments results in an increased cyclic fatigue resistance. In accordance with previous studies [28], regarding different reciprocation angles, for our study, we used 90° CW–30° CCW for the FRM study groups as a way to enhance the instrument resistance to fatigue failure.

One of the most important yet neglected factors that affects the cyclic fatigue resistance of endodontic instruments is the variation in the metallurgical characteristics of the evaluated NiTi systems [37]. Metallurgical tests are essential for fully comprehending the mechanical behavior of NiTi rotary instruments, especially in cases when comparing the performance of various endodontic instruments [38]. The present study’s results regarding the XRF analysis are consistent with those of previous studies [39,40], which reported that the alloying elements in the allotropic transformation of the NiTi alloy’s crystalline structure are divided into three categories: alphagenic elements, which stabilize the β phase, neutral elements, and betagenic elements, which stabilize the α phase. Although O, C, and N can be utilized, Al is the most significant alloying element among the alphagenic elements. The XRF analysis’s findings demonstrated the existence of the aforementioned alloying component in an almost equal percentage. In our study, the M3 files showed a more evident Al peak (Al—2.54%) compared to the PTU files (Al—1.74%,) and the PTG files (Al—1.40%,). The presence of Al results in a more martensitic crystalline structure, which, in turn, leads to a more flexible and fracture-resistant alloy. The cyclic fatigue resistance is also significantly impacted by body temperature [41,42]. Depending on the alloy’s prior heat treatment, the temperature at which the instruments are tested may have an impact on the results of the cyclic fatigue resistance of NiTi files, which exhibit distinct behavior [43]. The temperature of the surrounding environment can influence the martensitic transformation of the heat-treated NiTi alloy, the super-elastic behavior, and the cyclic fatigue resistance, which can shorten the fatigue life of an instrument exposed to body temperature [44]. Although it is known that the body temperature affects the cyclic fatigue resistance, this in vitro study was conducted at room temperature, taking into consideration the findings of Plotino in his 2017 [43] research, who found that, while the fatigue life of instruments made with gold heat treatment was unaffected, the intracanal temperature had an impact on the cyclic fatigue resistance of instruments made with conventional NiTi alloy. ProTaper instruments’ resilience to cycle fatigue is improved by gold heating treatment [42]. Within the limitations of this study, even if we also observed improved performance in the cyclic fatigue resistance for all the files tested when FRM was employed, we are in accordance with other studies, which stated that, depending on the proprietary heat treatment of the files, the cyclic fatigue testing could exhibit a different outcome [45], and we consider that further research is necessary to fully comprehend the effect of using FRM on the cyclic fatigue resistance of rotary instruments designed to use CR and expand the research to other instruments manufactured with different types of heat treatment.

The present study showed that all of the file systems had an improved TTF/NCF when FRM was employed, in accordance with the findings derived from V Faus-Matoses (2022) [29], Ferreira F et al. (2017) [46], and Van der Vyver 2020 [47], who indicated that the resistance of NiTi alloy endodontic rotary files to cyclic fatigue was better when reciprocating motion was used compared to CR.

Considering that the majority of instruments are single-use in order to prevent cross-contamination, cyclic fatigue does not play a major role in a clinical environment, as the number of rotations during clinical use is much lower than the limits measured in the cyclic fatigue tests [48]; as such, in accordance with Özyürek et al. 2018 [49], we can postulate that, while NCF is not the best way to assess the clinical performance of any one file, since during its clinical use it is hard to reach the number of rotations necessary to fracture the instrument, it is more of a mathematical analysis used in in vitro testing, as it is the best method to evaluate the metallurgical properties (elasticity and resistance to breakage) of the file itself.

Further in-depth research is necessary regarding the metal alloy and the elasticity modulus in order to fully comprehend the clinical implications and limitations of the tested files and the motion kinematics employed (FRM) in clinical practice.

## 5. Conclusions

The PTU files tested showed superior cyclic fatigue resistance over the PTG and M3 files regardless of the motion kinematics employed. The PTU files present a different cross section than the PTG and M3 but seem to have a similar surface finish and similar NiTi ratios. Under these experimental conditions, the reciprocating motion tested (i.e., FRM) considerably enhanced the cyclic fatigue resistance of all the rotary files tested compared with CR.

## Figures and Tables

**Table 1:** The mean and standard deviation of the TTF (s)/NCF (r).

		PTU		PTG		M3	
		CR	FRM	CR	FRM	CR	FRM
TTF (s)	Average	50	156	21	105	19	115
	St. deviation	7.6	21.6	3.3	7.2	1.27	8.4
NCF (r)	Average	333	1040	144	702	131	766
	St. deviation	51.2	144	22.4	48.1	8.4	56.2

s—seconds, r—rotations.

**Table 2:** Descriptive analysis of the TTF (s)/NCF (r) between the files using different motion kinematics.

Type of Motion × Type of File	Difference (s)	*p* Value
CR × PTU vs. CR × M3	30.329	<0.0001
CR × PTU vs. CR × PTG	28.478	<0.0001
CR × PTG vs. CR × M3	1.851	0.998707
FRM × PTU vs. FRM × PTG	50.778	<0.0001
FRM × PTU vs. FRM × M3	41.099	<0.0001
FRM × M3 vs. FRM × PTG	9.679	0.323732

s—seconds.

## Data Availability

The data from this research are available from the corresponding authors upon reasonable request. Data is not publicly available due to privacy. The present study is part of my Phd research which has not been publicly presented yet.
